# The cancer translational research informatics platform

**DOI:** 10.1186/1472-6947-8-60

**Published:** 2008-12-24

**Authors:** Patrick McConnell, Rajesh C Dash, Ram Chilukuri, Ricardo Pietrobon, Kimberly Johnson, Robert Annechiarico, A Jamie Cuticchia

**Affiliations:** 1 SemanticBits, Herndon, Virginia, USA; 2 Duke University Medical Center, Durham, North Carolina, USA; 3 Duke Bioinformatics Group, Duke Comprehensive Cancer Center, DUMC Box 2717, USA

## Abstract

**Background:**

Despite the pressing need for the creation of applications that facilitate the aggregation of clinical and molecular data, most current applications are proprietary and lack the necessary compliance with standards that would allow for cross-institutional data exchange. In line with its mission of accelerating research discoveries and improving patient outcomes by linking networks of researchers, physicians, and patients focused on cancer research, caBIG (cancer Biomedical Informatics Grid™) has sponsored the creation of the caTRIP (Cancer Translational Research Informatics Platform) tool, with the purpose of aggregating clinical and molecular data in a repository that is user-friendly, easily accessible, as well as compliant with regulatory requirements of privacy and security.

**Results:**

caTRIP has been developed as an N-tier architecture, with three primary tiers: domain services, the distributed query engine, and the graphical user interface, primarily making use of the caGrid infrastructure to ensure compatibility with other tools currently developed by caBIG. The application interface was designed so that users can construct queries using either the Simple Interface via drop-down menus or the Advanced Interface for more sophisticated searching strategies to using drag-and-drop. Furthermore, the application addresses the security concerns of authentication, authorization, and delegation, as well as an automated honest broker service for deidentifying data.

**Conclusion:**

Currently being deployed at Duke University and a few other centers, we expect that caTRIP will make a significant contribution to further the development of translational research through the facilitation of its data exchange and storage processes.

## Background

In order to have an impact in society, discoveries in cancer research need to be translated into knowledge that can be directly applied to treatment and prevention. These discoveries usually start within the basic sciences, from experiments developed at the molecular level, slowly progressing to clinical research. Although this translational process is at the very basis of our ability to generate new biomedical knowledge, to date few tools have been developed to successfully link the basic and clinical science fields in a way that researchers from both arenas can easily make connections. More specifically, cancer research would benefit from the development of applications that can aggregate clinical and molecular data in a repository that is user-friendly, easily accessible, as well as compliant with regulatory requirements of privacy and security.

In alignment with the requirements outlined above, the Duke Comprehensive Cancer Center (DCCC), in collaboration with SemanticBits LLC, has developed the Cancer Translational Research Informatics Platform (caTRIP, , this and all caBIG links last current as of July 2008), a translational research system for the Cancer Biomedical Informatics Grid (caBIG) project [1]. caTRIP allows users to query across a number of caBIG data services, join on common data elements (CDEs), and view their results in a user-friendly interface. Having outcomes analysis as its initial focus, caTRIP allows clinicians to query across data from existing patients with similar characteristics to find treatments that were administered with success. In doing so, caTRIP can help inform treatment and improve patient care, as well as enable searching for available tumor tissue, locating patients for clinical trials, and investigating the association between multiple predictors and their corresponding outcomes (such as survival). Of importance, caTRIP relies on the vast array of open source caBIG applications, including (1) the Tumor Registry, a clinical system that is used to collect endpoint data; (2) the cancer Text Information Extraction System (caTIES, ), a locator of tissue resources that works via the extraction of clinical information from free text surgical pathology reports while using controlled terminologies to populate caBIG-compliant data structures; (3) caTissue CORE , a tissue bank repository tool for biospecimen inventory, tracking, and basic annotation; (4) Cancer Annotation Engine (CAE ), a system for storing and searching pathology annotations; (5) caIntegrator ), a tool for storing querying, and analyzing translational data, including SNP data.

The objective of this article is to describe the caTRIP system in relation to its objectives, overall software architecture, security, implementation, usability, and future development plans.

### Case Study: Outcomes Analysis

A motivating use case for the development of caTRIP is one of outcomes analysis, whereby a clinician can query data from a cohort of preexisting patients to help guide treatment of another patient. An oncologist can ask "What are the treatments and outcomes of other patients that have similar characteristics to my patient?" caTRIP makes this possible on multiple levels: local, institutional, regional, national, and beyond by leveraging grid infrastructure to scale beyond traditional query systems.

Consider an example of a patient with a breast lump approaching an oncologist for treatment at a tertiary care facility. The patient has been told by her local physician that the tumor she has is not uncommon, but difficult to manage. The oncologist reviews the medical documents from the outside institution and recognizes an entity that is well-equipped to handle a lobular carcinoma of the breast. What is slightly odd is that this tumor has an intermediate nuclear grade and has been further designated as a "pleomorphic lobular carcinoma". The oncologist is familiar with the medical literature on the topic and recognizes that in certain circumstances such an entity may exhibit biologic behavior akin to an invasive ductal tumor (rather than that of a typical lobular). Paralleling the odd histologic finding is an additional finding that the tumor is negative for estrogen receptor (ER) expression, negative for progesterone receptor (PR) expression, but strongly positive for human epidermal growth factor receptor 2 (Her-2/neu) overexpression. This profile is very uncharacteristic for a lobular carcinoma of the breast. Thinking that the tumor may have been misclassified, the oncologist has the pathology material reviewed again and re-runs the molecular studies to confirm the receptor studies reported from the outside institution. To complicate matters, the reviewing pathologist reclassifies the tumor as a typical lobular carcinoma (not pleomorphic), but the receptor study profiles (ER, PR) and Her-2/neu status remain unchanged. Now there is no diagnostic correlate for the receptor profile results. Lobular tumors (particularly typical ones) are nearly always positive for ER and PR receptor (and usually strongly so) and negative for Her-2/neu overexpression.

**Figure 1:**
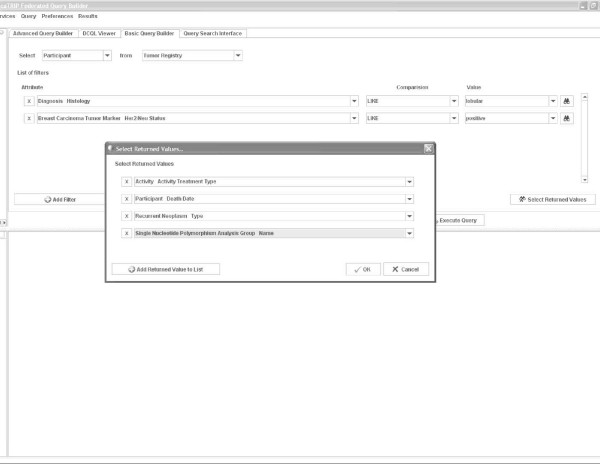
**Screenshot of the Simple Interface depicting a outcomes analysis translational query**.

As an example, at Duke, the Breast Pathology service will typically see one or two such cases per year *at most*. The expected biologic behavior and sensitivity to various treatment protocols are unknown in such cases. The body of literature on this particular scenario of findings is still maturing [2-5]. The fact of the matter is that even a large tertiary care facility will rarely come across such tumors. caTRIP provides a mechanism to examine all those patients who have been seen at any institution where caTRIP has been deployed and identify the cohort of patients that matches the specified criteria. It is a matter of a few minutes, at most, to construct a query that narrows the cohort to patients that have lobular tumors of the breast, are ER negative, PR negative, and Her-2/neu overexpressed (Figure 1). Furthermore, the type of treatment employed and the time to recurrence or death are easily captured as part of the result set by drawing upon the Tumor Registry as an additional data source. This permits a level of decision *in the clinic *– heretofore impossible with prior technologies. Running such a query at Duke returns very few cases because of the relative rarity of such clinical phenotypes. However, caTRIP is grid-enabled, and, if deployed at Cancer Centers across the country as part of the NCI's caBIG initiative, the statistical power could easily increase by multiple orders of magnitude. Rather than relying on single-institution or limited data sets published in the literature, an oncologist now has access to a rich *live *data set that can provide strong statistically significant data in mere minutes.

While the preceding scenario focuses on facilitating clinical care, caTRIP is adept at enabling research as well. Consider a follow-up situation in which a researcher would like to take that cohort of patients that have lobular carcinoma and Her-2/neu overexpression and compare them to a cohort that have ductal tumors that also overexpress Her-2/neu, as well as to a third group that have lobular tumors but not overexpress Her-2/neu. DNA arrays can be used to test for the presence of hundreds or even thousands of gene sequences in relatively short order if tissue samples are available. caTRIP can cross-query linked biospecimen repositories to identify samples that are available for the three cohorts of patients and retrieve samples that match specified criteria (e.g. frozen diseased tissue with match adjacent normal tissue). With informatics solutions like caTRIP and newer molecular technologies like DNA arrays, the time and effort to develop new diagnostic and prognostic markers of disease can be significantly reduced.

### Design Objectives

The objectives and sample questions that caTRIP is designed to address include:

1. Allow for the search of available tumor tissue for patients with factors of interest. A typical question to be answered by caTRIP would thus be: "*What are all the tissue specimens from Her-2/neu positive patients that have a primary tumor in the breast and are BRCA1 positive?"*

2. Identify predictors contributing to increased or decreased survival among a subset of cancer patients. caTRIP should answer questions such as: *"What are all the ER positive patients that have survived breast cancer after radiation treatment?"*

3. Determine the distribution over time of a number of factors that may contribute to or reflect progression of a disease state: "*Does a change in pathology biomarkers over time contribute to recurrence or death?*"

4. Determine the association between a multitude of clinical predictors and their respective post-operative outcomes, such as *"Does a change in ER or PR status before and after surgery correlate with other factors?"*

5. Identify patients eligible for participation in clinical trials that meet a pre-defined set of inclusion and exclusion criteria, such as "*List all patients that are triple negative (ER, PR, and Her-2/neu negative)."*

6. Identify pathology reports of interest, such as "*Show me all pathology reports for Her-2/neu positive patients with a lobular carcinoma*."

In addition, it is important that the system provide security and confidentiality levels in accordance with HIPAA (Health Insurance Portability and Accountability Act) and part 11 FDA regulations.

## Implementation

### Technical Requirements

Our high level scientific use cases were in part motivated by the need to access a variety of data types stored in a number of data systems at Duke. The Medical Assistant on the World Wide Web (MAW3), data from the Genetic Modifiers of BRCA 1/2 study (GEMS), and Tumor Registry data sources feed caTRIP pathology, clinical, tissue banking, single nucleotide polymorphism (SNP), and treatment/endpoint data. From this baseline, we defined the outcomes analysis use cases that feed into the system and application requirements that define the overall set of features that caTRIP implements (see Background for scientific use cases).

The system requirements primarily relate to development in the Cancer Biomedical Informatics Grid (caBIG) initiative [1]. caBIG has outlined a rigorous set of requirements related to interoperability that span the categories of legacy, bronze, silver, and gold compatibility. Silver compatibility places requirements on both the data elements that are exposed to/by caTRIP, as well as the application programming interfaces (APIs). Data elements leveraged in caTRIP must be defined as common data elements (CDEs) registered in the Cancer Data Standards Repository (caDSR, ). Each CDE must have one or more semantic concepts registered in the Enterprise Vocabulary Service (EVS, ). This combination of defining the structure of the data, as well as the underlying semantic concepts describing the meaning of the data provide caTRIP with semantically rich information models to query upon. However, in order to query across different data systems, it is critical that the CDEs be harmonized. This allows for data to be aggregated and presented to the user, as well as provides common CDEs, or keys, to join on. One way to catalogue these foreign CDEs is through a Master Person Index, which would provide a common identifier to link patients across systems [6]. It is possible, though not without challenges, to identify and link patients within a single institution using an institutional medical record number (MRN). However, queries that join on patients across institutional boundaries can be quite challenging.

Compatibility of the APIs in caTRIP falls more squarely into caBIG gold compatibility, which has not fully been defined by the caBIG program as of yet. All data that is accessible to caTRIP must be exposed via caGrid data services [7]. This means that the data is queriable via a well-defined, standards-based interface that accepts queries via the caGrid Common Query Language (CQL), which describes the query in object-oriented terms corresponding to the registered CDEs. Finally, there are also system requirements related to security, which include authenticating users based on credentials and authorizing users to access data.

### Software architecture

**Figure 2:**
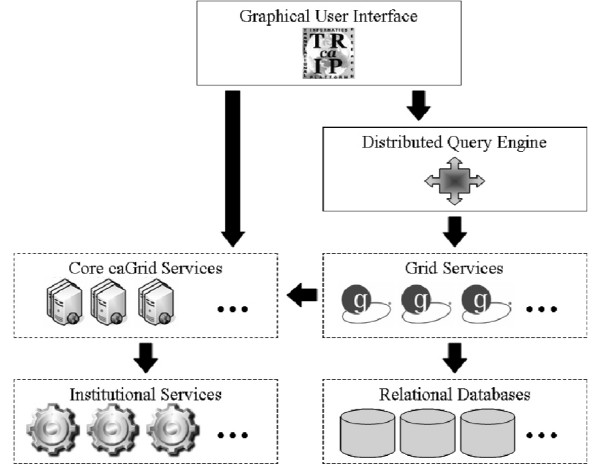
**High-level view of the caTRIP architecture, including Domain Services, Distributed Query Engine, and Graphical User Interface**.

caTRIP is developed as an N-tier architecture with three primary tiers: domain services, the distributed query engine (DQE), and the graphical user interface (Figure 2). The domain services are grid services that expose information models that encapsulate the underlying data. The distributed query engine takes as input a distributed common query language (DCQL) query, decomposes it into individual common query language (CQL) queries, issues those queries to the domain services, joins/aggregates the results, and returns the final results to the caller. The graphical user interface (GUI) provides a metadata-driven interface for building and running DCQL. It also provides an interface for saving and searching for queries.

Another important goal for caTRIP is to leverage existing technologies wherever possible. These include:

• *Globus 4.1 *for the underlying messaging stack

• *caGrid 1.0 *for the underlying service infrastructure

• *Introduce *for service creation

• *Index Service *for discovering services on the grid

• *Authentication Service *to authenticate users using their institutional credentials

• *Dorian *to obtain a grid user proxy

• *Grid Grouper *to authorize users to access data based on groups

• *Common Security Module (CSM) *for plugging authorization into the data service

• *Cancer Data Standards Repository (caDSR) *for storing common data elements (CDEs)

• *Enterprise Vocabulary Services (EVS) *for storing semantic concepts

• *Hibernate *for an object-relational mapping

• *Oracle *and *MySQL *for backend relational databases

More information can be found in the caTRIP architecture document and UML models:

• 

• 

### Implementation at Duke

We deployed caTRIP at Duke to leverage data from the Genetic Modifiers of BRCA1 and BRCA2 study in the Duke Breast Specialized Program of Research Excellence (SPORE). The goal of the GEMS investigation is to identify factors that influence the incidence of breast cancer in germline BRCA1/2 mutation carriers. To this end, we deployed tissue banking and pathology data from MAW3 into caTissue CORE, CAE, and caTIES; treatment, recurrence, and endpoint data from our Tumor Registry into a grid service; and SNP data from flat files into caIntegrator. These services were secured and then exposed to the caTRIP interface. This deployment allowed us to answer all of the domain questions that are found in the Design Objectives section of this article. Users of this pilot deployment included a set of pathologists and clinicians who provided feedback that guided the development, providing important feedback such as the use of menu-drive interfaces that require little knowledge of the backend data instead of drag-and-drop interfaces that do require such knowledge.

## Results and discussion

### Graphical User Interface

The Graphical User Interface (GUI) is a Java Swing-based thick client that can be accessed via Java WebStart . It provides the user with the ability to construct, execute, and share distributed queries in a graphical fashion. There are two primary ways to construct queries: the Simple Interface is a more limited, yet powerful way to build queries using drop-down menus, and the Advanced Interface is a flexible way to build queries using drag-and-drop.

### Simple Interface

**Figure 3:**
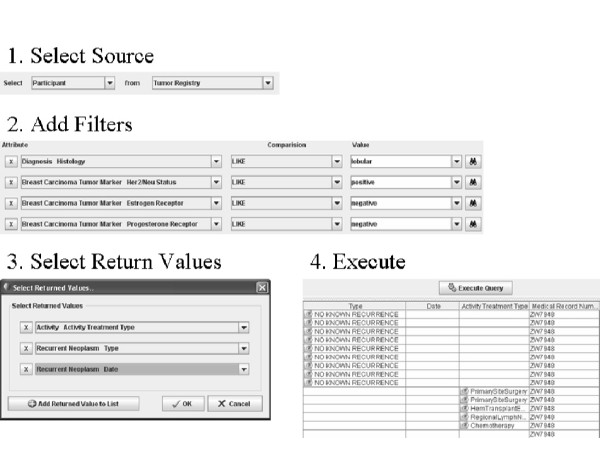
**Steps to perform a query in the caTRIP Simple Interface**.

**Figure 4:**
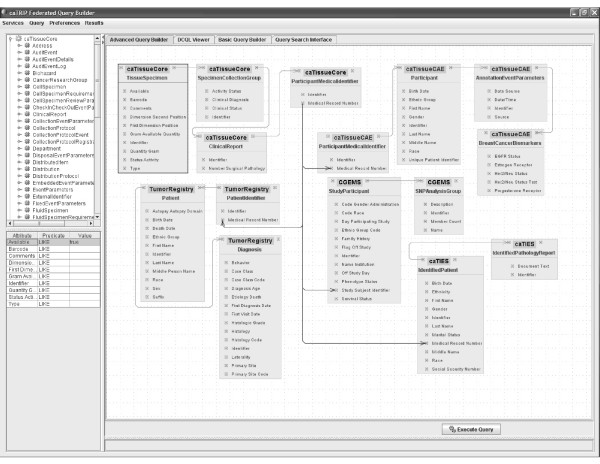
**Example Advanced Interfacing, depicting the potential complexity of distributed queries**.

The "Simple Interface" was developed to target the non-technical users such as a clinician. Queries are constructed using drop-down menus to add filters, AND/OR groups, and return attributes (Figures 1 and 3). Available services are selected via a configuration file, as well as the CDEs that make up the drop-down menu items for filters and return attributes. Joins across these services are performed using common identifiers. Specifically, in caTRIP, we use the (optionally) deidentified Medical Record Number (MRN) of patients to join and aggregate data. The data models of the underlying services are composed of potentially complex classes, attributes, and associations between the classes (see Figure 4). In order to construct queries that span multiple classes and associations, the Simple Interface leverages a configuration file that describes the outbound path from each class to the MRN, as well as the inbound path to each filterable/returnable CDE. This enables the automated logic that would normally be performed by a user manually selecting all of the associations necessary to perform a query.

### Advanced Interface

The "Advanced Interface" was developed to target a data technician who may wish to perform more complex queries. Queries are constructed by dragging classes onto a canvas, drawing associations between them, and adding filters along the way (Figure 4). Because the logic of how to traverse the information space is not built-in like it is with the "Simple Interface", users must manually create the association paths to the classes/attributes that they would like to filter on, as well as those they would like to perform cross-service foreign joins with. This allows users to perform queries not supported by the "Simple Interface" but adds an extra level of complexity.

### Distributed Query Engine

The Distributed Query Engine (DQE) provides the logic to perform a query across different caGrid data services. The caTRIP team developed a distributed query engine, which was later adopted into the caGrid 1.0 toolkit as the Federated Query Processor (FQP) module. Currently, the DQE is available for use by the general caBIG community and is currently being leveraged by the Cancer Bench to Bedside (caB2B) project . It takes as input a Distributed Common Query Language (DCQL) query, breaks it into separate Common Query Language (CQL) queries, issues those queries to the appropriate underlying data services, receives the results, performs joins, aggregates data, and returns the results. The DQE is built as a Java service and can be deployed as a grid service.

### Domain Services

The domain services themselves can be further divided into a number of tiers, including a relational database management system (RDBMS), Hibernate, Common Query Language processor , and grid service. Hibernate provides an object-oriented abstraction of the underlying relational database, exposing tables and columns as classes and attributes in a flexible, configurable manner . The CQL processor translates a CQL query based upon common data elements and their associations into a Hibernate Query Language (HQL) query. This is a relatively complex mapping because CQL and HQL have much different syntaxes. Finally, the grid service provides a standards-based entry point to issue a CQL query. caGrid  provides the underlying technology stack for the grid service, which itself is based upon Globus 4.1 .

### Security

**Figure 5:**
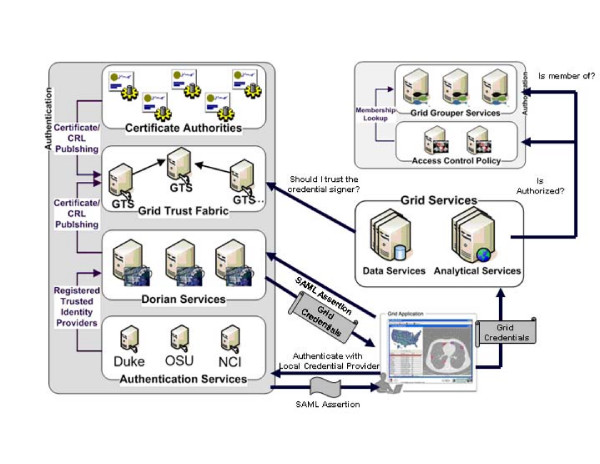
**High-level overview of the caGrid security architecture **.

caTRIP leverages the caGrid 1.0 infrastructure, which provides robust security for a distributed grid environment (Figure 5).

### Security: Authentication

The first step in the security workflow is authentication, which is the process by which a trusted organization asserts a user is who they claim to be. This is implemented using the caGrid Authentication Service. The goal is for the user to obtain a grid user certificate to be used in authorization. The user submits their credentials (user name and password) to the AuthenticationService, which has an institutional identity provider (IdP) plug-in. This component performs the necessary steps to determine that the user's credentials are valid. In the case of the Duke IdP plugin, the user name and password are validated against a Duke Domain Controller, which uses NT Security. This means that a user can log in with the same Cancer Center credentials they use to log in to their workstation. Once validated, the AuthenticationService returns a Security Assertion Markup Language (SAML) document  to the user, which asserts that the user is who they claim to be. The SAML Assertion can then be passed off to a Dorian Service to generate a grid user certificate. All of these interactions are handled for the user by the caTRIP application.

### Security: Authorization

The second step in the security workflow is authorization, which is the process by which a system grants the user access to specific resources. This is implemented using the Common Security Module (CSM) plug-in to the grid data service. The CSM {Phillips, 2006} is an open source toolkit that provides a flexible way to provision users and make authorization decisions. When a data field is accessed on behalf of the user, an authorization structure is queried to determine whether the user has permission to access it. If not, then a security exception is returned to the client. Authorization is made possible because trust exists between the service serving the data resource and the AuthenticationService.

### Delegation and Foreign CDEs

An alternate flow in the security workflow is delegation, which is the process by which a service acts on behalf of the user. This impacts caTRIP because the distributed query engine can be run as a service, so it must query the domain services using the user's certificate. The final implementation will likely leverage the DelegationService from the Globus project. The user will signal the DelegationService that it is permissible for a collocated service to act on the user's behalf. In the meantime, the distributed query engine is collocated with graphical user interface, so it simply passes the user's credentials to the data services identified in the distributed query. This bypasses some of the business rules defined by delegation, such as the number of delegation hops allowed by the user, but it is sufficient to meet caTRIP use cases.

Another change on the security workflow is the use of "foreign CDEs" for the purpose of joining data. It is conceivable that a user does not have permission to view a CDE that is used for joining between two different domain services. The classic example of this is to join patients across services using the Medical Record Number. In this case, a special security workflow must be undertaken during authorization. A level of trust is maintained between the distributed query engine service and the domain service such that foreign CDEs are returned to the DQE service with the understanding that the delegated user is not authorized to view them but is authorized to join on them. This workflow is only possible if the DQE is not collocated with the client application.

### Automated Honest Broker Service

The primary goal of caTRIP is to provide users access to diverse datasets that cross domains and data sources. One challenge that arises from this is deidentifying datasets that are managed by different groups, which especially affects deidentifying foreign CDEs (MRNs) that are used to link across different datasets. A typical scenario for cross-dataset deidentification is for an IRB appointed individual to collect each dataset and deidentify them all at once, tossing away the keys to the Protected Health Information (PHI). This approach is not scalable in a distributed environment because it requires a middle-man to perform the deidentification. Whenever a new dataset is added to the system, all previously existing data sets must be deidentified again. To address this in caTRIP, we developed an Automated Honest Broker Service (AHBS), which performs the tasks once performed by a human appointed by the Institutional Review Board. A data owner programmatically submits PHI (e.g. an MRN) to the AHBS using a secure connection. The AHBS generates a random key, associates it internally in a database to the PHI (which can be encrypted), and then returns the key to the data owner. The data owner then can deidentify his dataset by replacing the PHI with the random key. When more than one data owner/dataset is involved, the AHBS will always return the same random key for the same PHI. In this way, diverse, distributed datasets can be deidentified using the same keys.

### Example from the Duke Implementation

The following example illustrates how caTRIP would construct and execute a user query that joins disparate datasets. A user at Duke could ask the question, "What are all of the available tissue specimens from the breast obtained from patients tested for positive expression of the estrogen receptor?" The distributed query engine would first fetch all of the patient medical record numbers from the Clinical Annotation Engine (CAE) service that have estrogen receptor expression annotated as positive. It would then issue a query to the caTissue CORE service for tissue specimens that are marked available, were collected from the tissue site of breast, and were obtained from patients that have medical record numbers that match those returned from the previous query. The results would be a list of tissue specimens, including their barcodes, location, size, etc. The single query issued to the distributed query engine would look like this:

1    <DCQLQuery xmlns = "">

2       <TargetObject name = "" serviceURL = ">

3          <Group logicRelation = "AND">

4             <Attribute name = "available" predicate = "EQUAL_TO" value = "true"/>

5             <Association name = "" roleName = "specimenCharacteristics">

6                <Attribute name = "tissueSite" predicate = "LIKE" value = "breast%"/>

7             </Association>

8             <Association name = "" roleName = "specimenCollectionGroup">

9                <Association name = "" roleName = "clinicalReport">

10                   <Association name = "" roleName = "participantMedicalIdentifier">

11                      <ForeignAssociation>

12                         <JoinCondition>

13                            <LeftJoin>

14                               <Object>edu.wustl.catissuecore.domainobject.impl.ParticipantMedicalIdentifierImpl</Object>

15                               <Property>medicalRecordNumber</Property>

16                            </LeftJoin>

17                         <RightJoin>

18                               <Object>edu.duke.catrip.cae.domain.general.ParticipantMedicalIdentifier</Object>

19                               <Property>medicalRecordNumber</Property>

20                            </RightJoin>

21                      </JoinCondition>

22                      <ForeignObject name = "" serviceURL = "">

23                            <Association name = "" roleName = "participant">

24                               <Association name = "" roleName = "annotationEventParametersCollection">

25                                  <Association name = "" roleName = "annotationSetCollection">

26                                     <Attribute name = "estrogenReceptor" predicate = "LIKE" value = "POSITIVE%"/>

27                                  </Association>

28                            </Association>

29                         </Association>

30                      </ForeignObject>

31                   </ForeignAssociation>

32                </Association>

33          </Association>

         </Association>

      </Group>

   </TargetObject>

</DCQLQuery>

Note on line 1 that the query is rooted at caTissue CORE, where available specimens are filtered on line 4 and specimens from the tissue site of "breast" are filtered on line 6. Lines 11–31 define the foreign association between caTissue CORE and CAE. The medical record numbers (MRNs) of each service are joined on lines 12–21 by defining the left join criteria to be the MRN from caTissue CORE and the right join criteria to the be MRN from CAE. Line 22 selects the foreign service to be CAE, from which patients with estrogen receptor expression marked as positive are filtered on line 26. The query itself is unfolded and executed inside-out by the distributed query engine, CAE being the first service to be queried and the results used to query caTissue CORE. Note also that the association paths of the objects are defined within the query on lines 5, 8, 9, 10, 23, 24, and 25, the logic of which is hidden from the users who are simply selecting data elements to query upon and return. The abbreviated results would look like this:

1    <queryResults targetClassname = "">

2    <ns1:ObjectResult xmlns:ns1 = "">

3    <ns2:TissueSpecimen id = "397" type = "Fixed Tissue Block" available = "true" positionDimensionOne = "20" positionDimensionTwo = "22" barcode = "ISBN-10 AB00-0013XY" comments = "TISSUE IN FORMALIN 30" activityStatus = "Active" quantityInGram = "0.7" availableQuantityInGram = "0.199999988079071" xmlns:ns2 = ""/>

4    </ns1:ObjectResult>

5    <ns2:ObjectResult xmlns:ns2 = "">

6    <ns3:TissueSpecimen id = "406" type = "Fixed Tissue Block" available = "true" positionDimensionOne = "19" positionDimensionTwo = "14" barcode = "ISBN-10 AB00-0043XY" comments = "STRONG FAMILY HISTORY FOR BRCA MUTATIONS" activityStatus = "Active" quantityInGram = "6.75" availableQuantityInGram = "6.25" xmlns:ns3 = ""/>

7    </ns2:ObjectResult>

8    <ns3:ObjectResult xmlns:ns3 = "">

9    <ns4:TissueSpecimen id = "411" type = "Fixed Tissue Block" available = "true" positionDimensionOne = "21" positionDimensionTwo = "7" barcode = "ISBN-10 AB00-0063XY" comments = "NO MORE WHOLE BLOOD" activityStatus = "Active" quantityInGram = "42.75" availableQuantityInGram = "42.25" xmlns:ns4 = ""/>

10    </ns3:ObjectResult>

...

11    </queryResults>

Note that line 1 defines the type of object being returned in the query results, and that the results themselves found on lines 3, 6, and 9 are each of the objects that match the query criteria with data elements of interest embedded within them as attributes.

## Conclusion

caTRIP is being implemented in a phased-development cycle, meeting certain objectives at each point to provide utility as the program is extended. It is currently in its second phase of development. Phase one concluded the pilot development activities of caTRIP, providing a baseline architecture and proof-of-concept user interface. End user training was provided on a limited basis for the small multidisciplinary breast oncology group using live demonstrations and basic instructions provided through e-mail messages. Feedback was collected through e-mail as well. The multidisciplinary group was given direct access to the primary software developers and technical experts during this period. In phase two, we plan to deploy caTRIP-II along with enhancements determined through end user interaction. Furthermore, we are also in the process of engaging different groups at Duke to adopt caTRIP to help meet their research goals.

In phase one of caTRIP, use cases focused on intra-institutional problems, tearing down the silos of data-oriented systems in a single cancer center. In future iterations we plan to extend caTRIP to tackle cross-institutional use cases. This involves extending the caTRIP interface and distributed query engine to support aggregation of data from services that expose common data elements. For example, a motivating use case amongst prospective adopters is to aggregate treatment, endpoint, pathology, and tissue banking data from a number of different cancer centers. This will allow researchers and clinicians to expand their queries and answer questions that they previously could not at their own institution. Furthermore, we plan to integrate caTRIP with analytics, specifically focusing on microarray analysis. The caBIG gene expression database, caArray, is a rich source of central or distributed gene expression data, which itself can be tied to clinical data, such as pathology biomarkers. This microarray data can be analyzed and visualized dynamically using services provided by other caBIG applications, such as geWorkbench {Califano 2006 }, GenePattern [8], and Bioconductor[9]. This notion of building workflows based upon queries designed using caTRIP's simple interface can provide a powerful tool for both researchers and clinicians. Finally, we also plan to enhance the user interface, providing more sophisticated reporting and data mining functionality. All of these features will be driven by end-user requirements of the adopter sites, which is a critical facet to the caBIG initiative.

## Availability and requirements

*Project name*: Cancer Translational Research Informatics Platform (caTRIP)

*Project home page*: 

*Operating system(s)*: platform independent

*Programming language*: Java

*Other requirements*: Tomcat 5.0.28, a database (MySQL 4.1.10 or Oracle 10.x), Java Version 1.5 or higher, and Apache Ant version 1.6.5

*License*: NCI caBIG license (non-viral)

*Any restrictions to use by non-academics*: no

## Competing interests

The authors declare that they have no competing interests.

## Authors' contributions

PM was the Lead Architect and Project Manager of caTRIP. RD provided subject matter expertise to guide the development of caTRIP. Ram Chilukuri was the lead developer of caTRIP. RP provided subject matter expertise. KJ provided subject matter expertise. RA was the project director. AJC was the project PI.

## Pre-publication history

The pre-publication history for this paper can be accessed here:


